# Inhibition of pyroptosis and apoptosis by capsaicin protects against LPS-induced acute kidney injury through TRPV1/UCP2 axis *in vitro*

**DOI:** 10.1515/biol-2022-0647

**Published:** 2023-07-29

**Authors:** Jinrun Han, Jinhao Wu, Hong Liu, Yu Huang, Wen Ju, Yifei Xing, Xiaoping Zhang, Jun Yang

**Affiliations:** The Department of Urology, Union Hospital, Tongji Medical College, Huazhong University of Science and Technology, Wuhan, Hubei, China; The Intensive Care Unit, Union Hospital, Tongji Medical College, Huazhong University of Science and Technology, Wuhan, Hubei, China; The Department of Urology, Union Hospital, Tongji Medical College, Huazhong University of Science and Technology, No. 1277 Jiefang Avenue, Wuhan, Hubei, China

**Keywords:** acute kidney injury, capsaicin, TRPV1, UCP2, pyroptosis, apoptosis

## Abstract

Acute kidney injury is a fatal disease characterized by a rapid deterioration of kidney function. Capsaicin (*trans*-8-methyl-*N*-vanillyl-6-nonenamide) is a natural product extracted from *Capsicum*. The aim of this study was to explore the protective effect of capsaicin on inflammation, apoptosis, and mitochondrial dysfunction in an *in vitro* model of acute kidney injury. Lipopolysaccharide (LPS)-induced acute kidney injury model was established in HK-2 cells to investigate the protective effect of capsaicin. Cell viability was assessed using CCK-8 assay, and protein expression was detected using western blot and immunofluorescence assay. Intracellular reactive oxygen species (ROS) level and mitochondrial membrane potential were analyzed by flow cytometry. Cell apoptosis was detected by propidium iodide staining. The results showed that capsaicin ameliorated LPS-induced cytotoxicity *in vitro* and attenuated the release of interleukin (IL)-1β and IL-18. Intriguingly, genipin abolished the protective effect of capsaicin. Molecularly, capsaicin activated transient receptor potential cation channel subfamily V member 1 –mitochondrial uncoupling protein 2 axis and inhibited caspase-1-mediated pyroptosis. In addition, capsaicin alleviated LPS-induced ROS production and mitochondrial membrane potential disruption and inhibited apoptosis. These findings suggest that capsaicin shows a protective effect in *in vitro* acute kidney injury model.

## Graphical abstract

**Figure:**
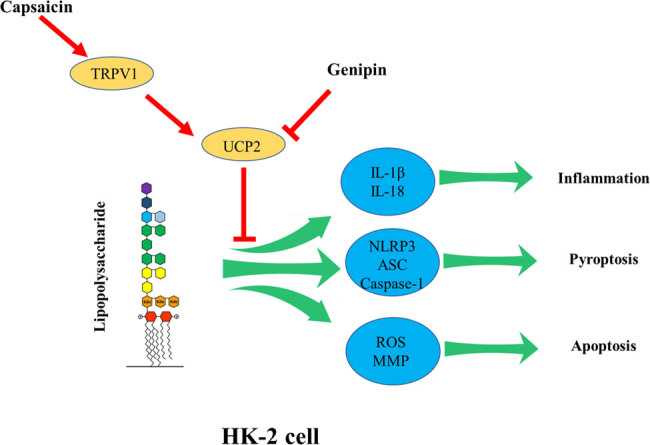

## 1 Introduction

Acute kidney injury is a serious condition characterized by the rapid deterioration of kidney function that could exacerbate in a few hours or days [1]. Common causes of acute kidney injury include lack of blood flow, drugs that damage kidney function, and infection [2,3]. Notably, sepsis that resulted from infection accounts for more than 50% cases of acute kidney injury [4]. Oxidative stress, inflammatory response, and programmed cell death (PCD) are closely associated with the pathogenesis of sepsis-related acute kidney injury [5,6]. Lipopolysaccharides (LPSs) are amphipathic glycoconjugates that can be isolated from the outer membrane of gram-negative bacteria and are commonly used to establish the acute kidney injury model *in vitro* [7]. Unfortunately, there is no established remedy for acute kidney failure, which results in poor outcomes and high mortality [8]. Hence, it is of great urgency to explore and develop efficient drugs for the clinical treatment of acute kidney injury.

Capsaicin (*trans*-8-methyl-*N*-vanillyl-6-nonenamide) is extracted from chili peppers in the *Capsicum* genus [9]. Originally used as food additive, capsaicin is now widely applied as pharmaceutical reagent in various diseases [10]. Studies have shown that capsaicin has strong anti-inflammatory and anti-oxidant effects [11]. Additionally, the anti-cancer effect of capsaicin has been extensively investigated over the past decades [12]. However, capsaicin was shown to be a double-edged sword in cancer treatment, with high concentrations inducing apoptotic cell death in a variety of cancer cells and low concentrations enhancing invasive and migratory ability of cancer cells [13]. Transient receptor potential cation channel subfamily V member 1 (TRPV1) is part of the mammalian somatosensory system, and capsaicin-induced activation of TRPV1 has shown beneficial effects in diabetes, obesity, and liver injury [14,15]. In addition, the activation of TRPV1 protects the heart against apoptosis through PI3K/Akt pathway in ischemia/reperfusion injury [16]. However, whether capsaicin has a therapeutic effect in sepsis-related acute kidney injury remains to be investigated.

PCD is a controlled biological process, including apoptosis, pyroptosis, autophagy, and other non-canonical cell death [17]. Apoptosis, which is mediated by sequential cleavages of the aspartate-specific proteases, is the earliest characterized PCD [18]. Excessive apoptosis has been implicated in neurodegenerative diseases, ischemic heart disease, and sepsis [19]. Pyroptosis is an inflammatory type of PCD mediated by caspase-1 [20]. Apoptosis-associated speck-like protein containing a caspase recruitment domain (ASC) is an adapt protein and the recruitment of ASC to caspase-1 by NLR family pyrin domain containing 3 (NLRP3) is essential for the activation of pyroptosis [21]. Pyroptosis is often associated with inflammatory diseases such as colitis, pneumonia, and arthritis [22]. Previous studies have indicated that pyroptosis is involved in acute kidney injury [23]. Moreover, an anti-apoptotic effect of capsaicin has been suggested in several studies [24,25]. Therefore, targeting pyroptosis and apoptosis by capsaicin is a promising strategy for the clinical treatment of acute kidney injury.

This study aimed to explore the protective effect of capsaicin on inflammation, apoptosis, and mitochondrial dysfunction in an *in vitro* model of acute kidney injury. From the results, the authors propose the potential therapeutic use of capsaicin as a novel treatment strategy for acute kidney injury.

## 2 Methods

### 2.1 Cell culture

HK-2 cell line was cultured with Dulbecco’s modified Eagle’s medium supplemented with 10% fetal bovine serum at 37°C with 5% CO_2_. To establish *in vitro* acute kidney injury model, HK-2 cells were treated with 5 mM adenosine triphosphate (ATP) and 500 ng/mL LPS for 24 h. In some experiments, cells were preincubated with 100 µM capsaicin or with 100 µM capsaicin and 25 µM genipin for 8 h prior to the addition of LPS.

### 2.2 Cell viability assay

HK-2 cells were seeded in 96-well plate at the density of 1 × 10^4^ cells per well and treated with indicated reagents for 24 h. Then, the number of viable cells was detected with Cell Counting Kit-8 (#C0038, Beyotime Biotechnology) according to the manufacturer’s instructions.

### 2.3 Lactate dehydrogenase (LDH) activity assay

HK-2 cells were seeded in six-well plate and treated with indicated reagents for 24 h. The cells were then harvested and washed with phosphate-buffered saline (PBS) twice. LDH activity was detected using a commercial testing kit purchased from Nanjing Jiancheng Bioengineering Institute (#A020-2).

### 2.4 Western blot

Cells were lysed with radioimmunoprecipitation assay lysis buffer (#AS1004, ASPEN), and the protein concentration was detected using bicinchoninic acid protein assay kit (#AS1086, ASPEN). Total proteins were separated on 12% sodium dodecyl-sulfate polyacrylamide gel electrophoresis gel and transferred to PVDF membranes. The PDVF membranes were blocked with 5% bovine serum albumin and incubated with the following antibodies at 4°C overnight: IL-1β antibody (#16806-1-AP, Proteintech Group, Inc.), IL-18 antibody (#ab2073244, Abcam), TRPV1 (#66983-1-lg, Proteintech Group, Inc.), uncoupling protein 2 (UCP2) (#11081-1-lg, Proteintech Group, Inc.), cleaved caspase-1 (#AF4005, Affinity Biosciences), ASC (#ab283684, Abcam), NLRP3V (#15101, Cell Signaling Technology), and GAPDH antibody (#ab181602, Abcam). The membranes were then incubated with horseradish peroxidase-conjugated secondary antibody (#AS1107, ASPEN) for an hour at room temperature. Protein bands were acquired with chemiluminescence detection kit (#AS1059, ASPEN) and digital scanning system (#LiDE110, Canon).

### 2.5 Immunofluorescence assay

HK-2 cells were seeded in a six-well plate and treated with indicated reagents. The cells were washed twice with PBS, fixed in 4% paraformaldehyde, and then permeabilized with PBS containing 0.1% Triton X-100. For immunostaining, the cells were incubated with indicated primary antibody overnight and then incubated with CoraLite488-conjugated goat anti-rabbit IgG (H + L) (#SA00013-2, Proteintech Group, Inc.). Nucleus was stained with Hoechst33342 (#B8040, SolarBio Life Sciences). Images were obtained using an immunofluorescence microscope (Eclipse Ci-L, Nikon). For the quantification of immunofluorescence staining, 8-bit binary threshold images from the original panoramic immunofluorescence image were made, and the mean gray value was acquired using ImageJ.

### 2.6 Cell apoptosis assay

HK-2 cells were seeded in a six-well plate and treated with indicated reagents. The cells were then harvested and washed twice with PBS. Next, the cells were incubated with propidium iodide (#ST511, Beyotime Biotechnology) and Hoechst33342 (#B8040, SolarBio Life Sciences). Images were obtained using an immunofluorescence microscope (Eclipse Ci-L, Nikon).

### 2.7 Detection of intracellular reactive oxygen species (ROS) level

HK-2 cells were seeded in a six-well plate and treated with indicated reagents. The cells were then washed twice with PBS and incubated with ROS Assay Kit (#S0033, Beyotime Biotechnology). Intracellular ROS level was then detected with a flow cytometry (CytoFLEX, Beckman).

### 2.8 Detection of mitochondrial membrane potential

HK-2 cells were seeded in a six-well plate and treated with indicated reagents. The cells were then washed twice with PBS and stained using mitochondrial membrane potential assay kit (#C2006, Beyotime Biotechnology). Subsequently, the cells were processed with a flow cytometry (CytoFLEX, Beckman).

### 2.9 Statistical analysis

Data were presented as means ± standard deviation (SD), and one-way analysis of variance was used to determine statistical significance between groups. *p* < 0.05 was considered statistically significant.

## 3 Results

### 3.1 Capsaicin ameliorates LPS-induced cytotoxicity and inflammation response in HK-2 cells

**Figure 1:**
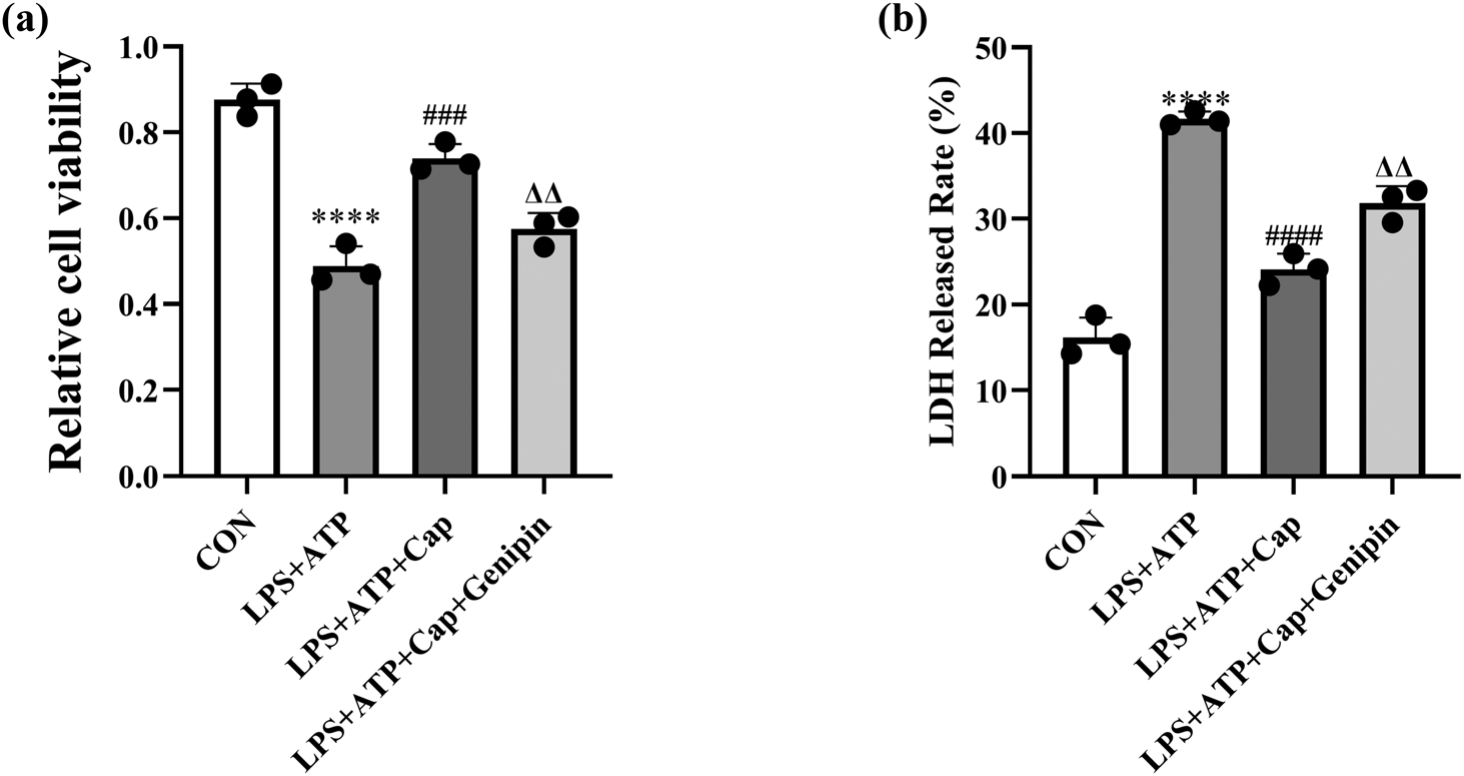
Capsaicin ameliorates LPS cytotoxicity in HK-2 cells. (a) Cell viability was detected by CCK8 assay in control, LPS + ATP, LPS + ATP + capsaicin and LPS + ATP + capsaicin + genipin groups. Data were presented as the means ± SD, ****p < 0.0001 vs CON, ###*p* < 0.001 vs LPS + ATP + capsaicin, ΔΔ*p* < 0.01 vs LPS + ATP + capsaicin + genipin. (b) LDH released rate was detected by the LDH activity assay kit. Data were presented as means ± SD, ****p < 0.0001 vs CON, ####*p* < 0.0001 vs LPS + ATP, ΔΔ*p* < 0.01 vs LPS + ATP + capsaicin.

**Figure 2:**
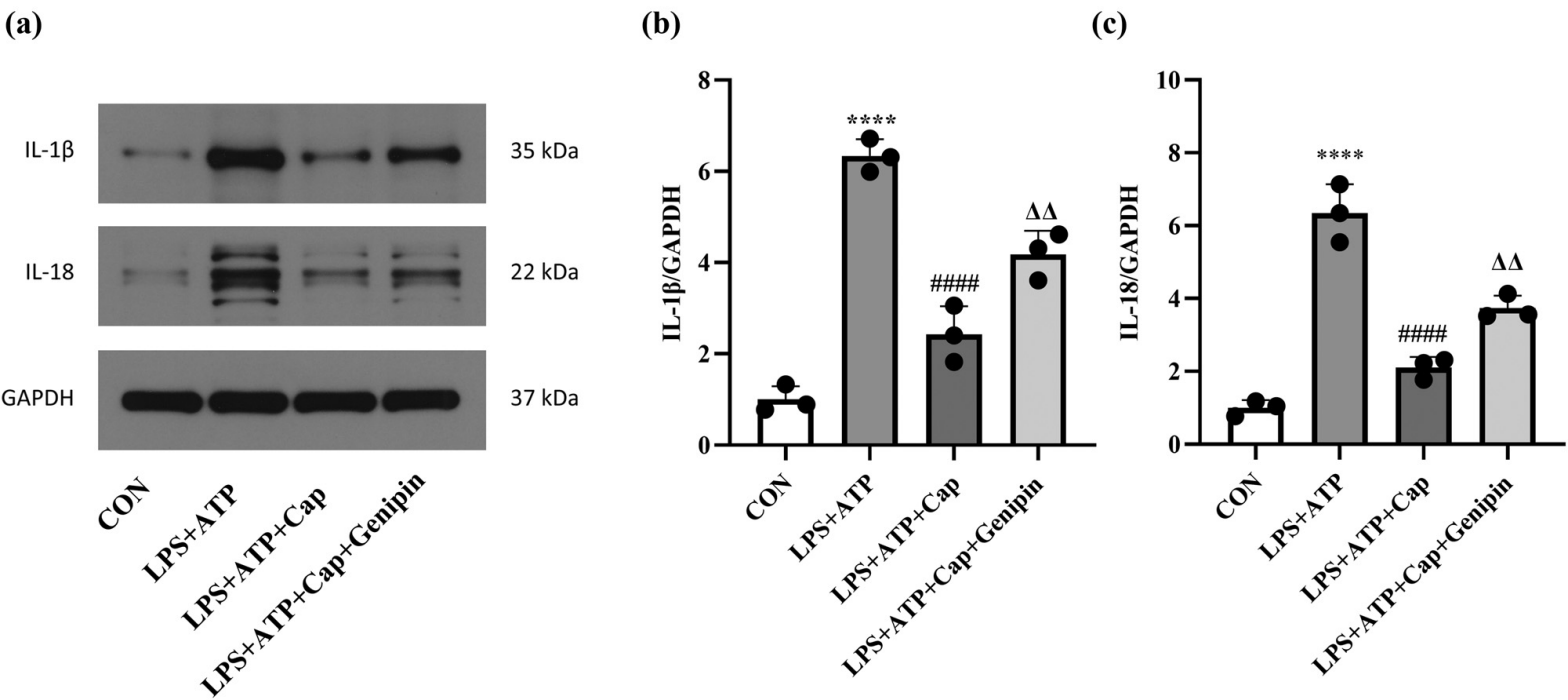
Capsaicin inhibits cytokine release in LPS-stimulated HK-2 cells. (a) Western blot results of IL-1β and IL-18 in control, LPS + ATP, LPS + ATP + capsaicin, and LPS + ATP + capsaicin + genipin groups. (b) and (c) Quantification of IL-1β and IL-18 expression in each group. Data were presented as the means ± SD, ****p < 0.0001 vs CON, ####*p* < 0.0001 vs LPS + ATP, ΔΔ*p* < 0.01 vs LPS + ATP + capsaicin.

An *in vitro* model of acute kidney injury was established by exposing HK-2 cells to LPS for 24 h. Dose incubation of LPS in HK2 cells differs in various reports ranging from 0.1 to 10 µg/mL [26,27,28]. A dose response to LPS and capsaicin in HK2 cells was performed in preliminary experiments. As shown in Figure 1a and Figure S1a, the exposure to LPS decreased the number of viable HK-2 cells in a dose-dependent manner, indicating successfully established *in vitro* model of acute kidney injury. Low concentrations of capsaicin had no effect on the viability of HK-2 cells (Figure S1b). To investigate the potential protective effect of capsaicin against LPS-induced acute kidney injury, HK-2 cells were pretreated with 100 μM capsaicin for 8 h and a CCK8 assay was performed. The results demonstrated that capsaicin ameliorated LPS cytotoxicity and the number of viable cells was restored. Activation of TRPV1/UCP2 axis by capsaicin has been reported in several disease models, including nonalcoholic fatty liver disease and diabetic cardiovascular complications [29,30]. To investigate whether similar mechanism was involved in LPS-induced injury model, genipin was used in combination with capsaicin. Interestingly, treatment with 25 μM genipin prevented the protective effect of capsaicin against LPS cytotoxicity. LDH release is a commonly used measurement that indicates the degree of cell death. As shown in Figure 2, an increased LDH release was observed in HK-2 cells following LPS treatment. Capsaicin treatment alleviated LPS-induced LDH release, which could be prevented by genipin. These results indicate that capsaicin has a potential protective effect against LPS-induced acute kidney injury.

Acute kidney injury is closely associated with inflammation response [31]. IL-1β and IL-18 are two inflammatory cytokines that regulate inflammation response at multiple checkpoints [32]. Consistent with previous studies, LPS treatment increased the release of IL-1β and IL-18 in HK-2 cells. However, capsaicin treatment attenuated the release of these cytokines. Notably, the inhibitory effect of capsaicin on LPS-induced expression of IL-1β and IL-18 was prevented by Genipin (Figure 2a and b). These data suggest that capsaicin alleviates LPS-induced inflammation response in HK-2 cells.

### 3.2 Capsaicin alleviates LPS-induced pyroptosis in HK-2 cells by activating TRPV1/UCP2 axis

**Figure 3:**
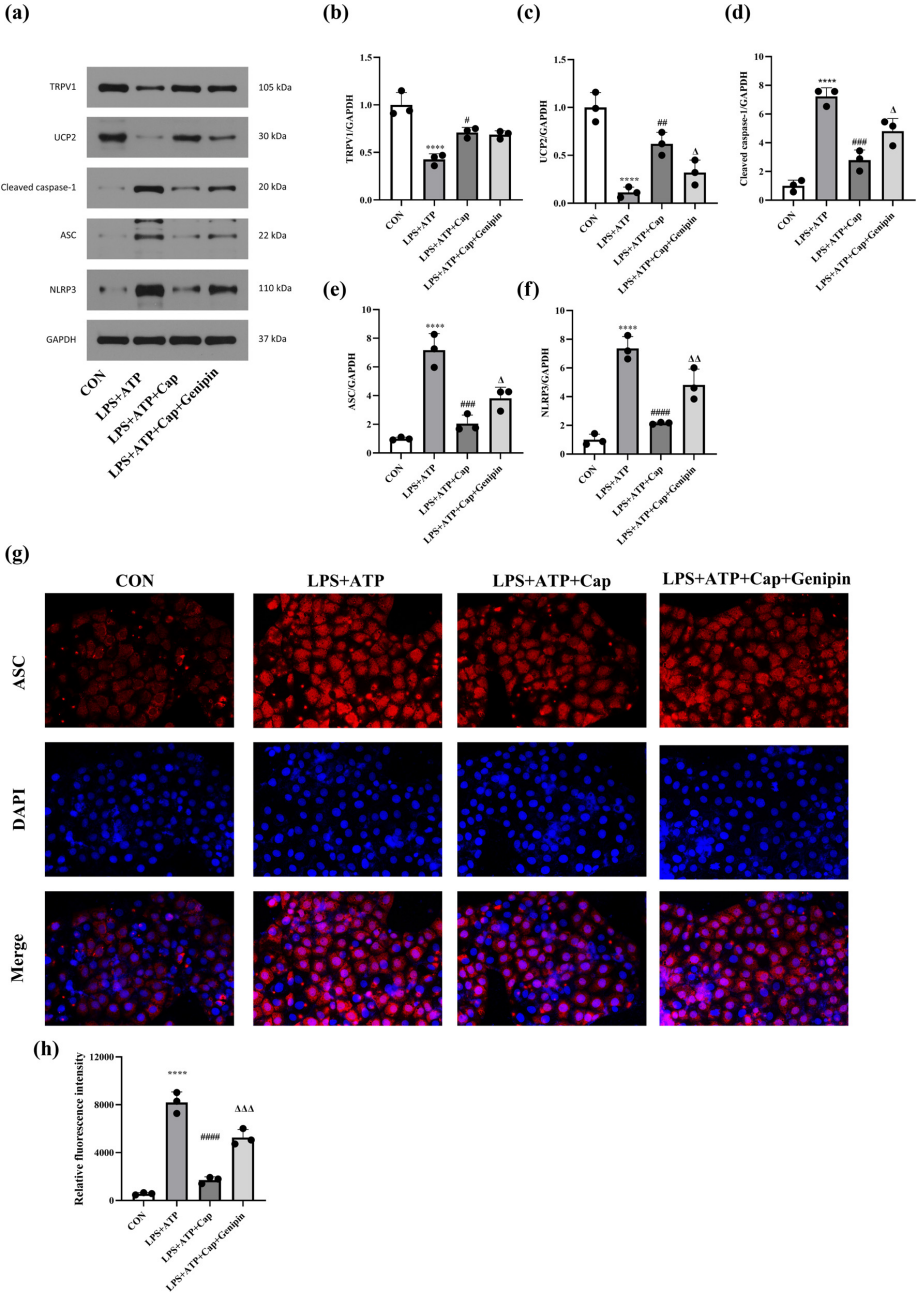
Capsaicin alleviates LPS-induced pyroptosis in HK-2 cells by activating TRPV1/UCP2 axis. (a) Western blot results of TRPV1, UCP2, cleaved caspase-1, ASC and NLRP3 in control, LPS + ATP, LPS + ATP + capsaicin, and LPS + ATP + capsaicin + genipin groups. (b) Quantification of TRPV1 expression in each group. Data were presented as means ± SD, ****p < 0.0001 vs CON, #*p* < 0.05 *vs* LPS + ATP. (c) Quantification of UCP2 expression in each group. Data were presented as means ± SD, ****p < 0.0001 vs CON, ##*p* < 0.01 vs LPS + ATP, Δ*p* < 0.05 vs LPS + ATP + capsaicin. (d) Quantification of cleaved caspase-1 expression in each group. Data were presented as means ± SD, ****p < 0.0001 vs CON, ###*p* < 0.001 vs LPS + ATP, Δ*p* < 0.05 vs LPS + ATP + capsaicin. (e) Quantification of ASC expression in each group. Data were presented as means ± SD, ****p < 0.0001 vs CON, ###*p* < 0.001 vs LPS + ATP, Δ*p* < 0.05 vs LPS + ATP + capsaicin + genipin. (f) Quantification of NLRP3 expression in each group. Data were presented as means ± SD, ****p < 0.0001 vs CON, ####*p* < 0.0001 vs LPS + ATP, ΔΔ*p* < 0.01 vs LPS + ATP + capsaicin. (g) Immunofluorescence results of ASC in each group. (h) Quantification of the fluorescence density of ASC in each group. Data were presented as the means ± SD, ****p < 0.0001 vs CON, ####*p* < 0.0001 vs LPS + ATP, ΔΔΔ*p* < 0.005 vs LPS + ATP + capsaicin.

TRPV1 is a receptor of capsaicin, and genipin is an inhibitor of UCP2 [30]. This study investigated whether capsaicin protects against LPS-induced acute kidney injury through TRPV1/UCP2 axis. Western blot results showed that LPS treatment down-regulated the expression of TRPV1 and UCP2, which was restored by capsaicin. Moreover, genipin treatment prevented capsaicin-induced expression of UCP2 in LPS-treated HK-2 cells (Figure 3a–c).

Pyroptosis has recently been identified as a mechanism involved in LPS-induced acute kidney injury [23]. Therefore, we explored whether capsaicin protects against LPS-induced acute kidney injury by targeting pyroptosis. Results of western blot showed that the expression of cleaved caspase-1, ASC, and NLRP3 was up-regulated following LPS treatment, indicating the activation of pyroptosis. However, capsaicin treatment down-regulated the protein levels of cleaved caspase-1, ASC, and NLRP3, and this effect was prevented by genipin (Figure 3d–f). Moreover, ASC expression was determined by immunofluorescence assay. As shown in Figure 3g and h, fluorescence density increased in the LPS-treated group compared with that in the control group. Capsaicin treatment resulted in a decreased fluorescence density of ASC, consistent with western blot results. All these data indicate that capsaicin alleviates LPS-induced pyroptosis in HK-2 cells by activating TRPV1/UCP2 axis.

### 3.3 Capsaicin attenuates LPS-induced ROS generation and apoptosis in HK-2 cells

**Figure 4:**
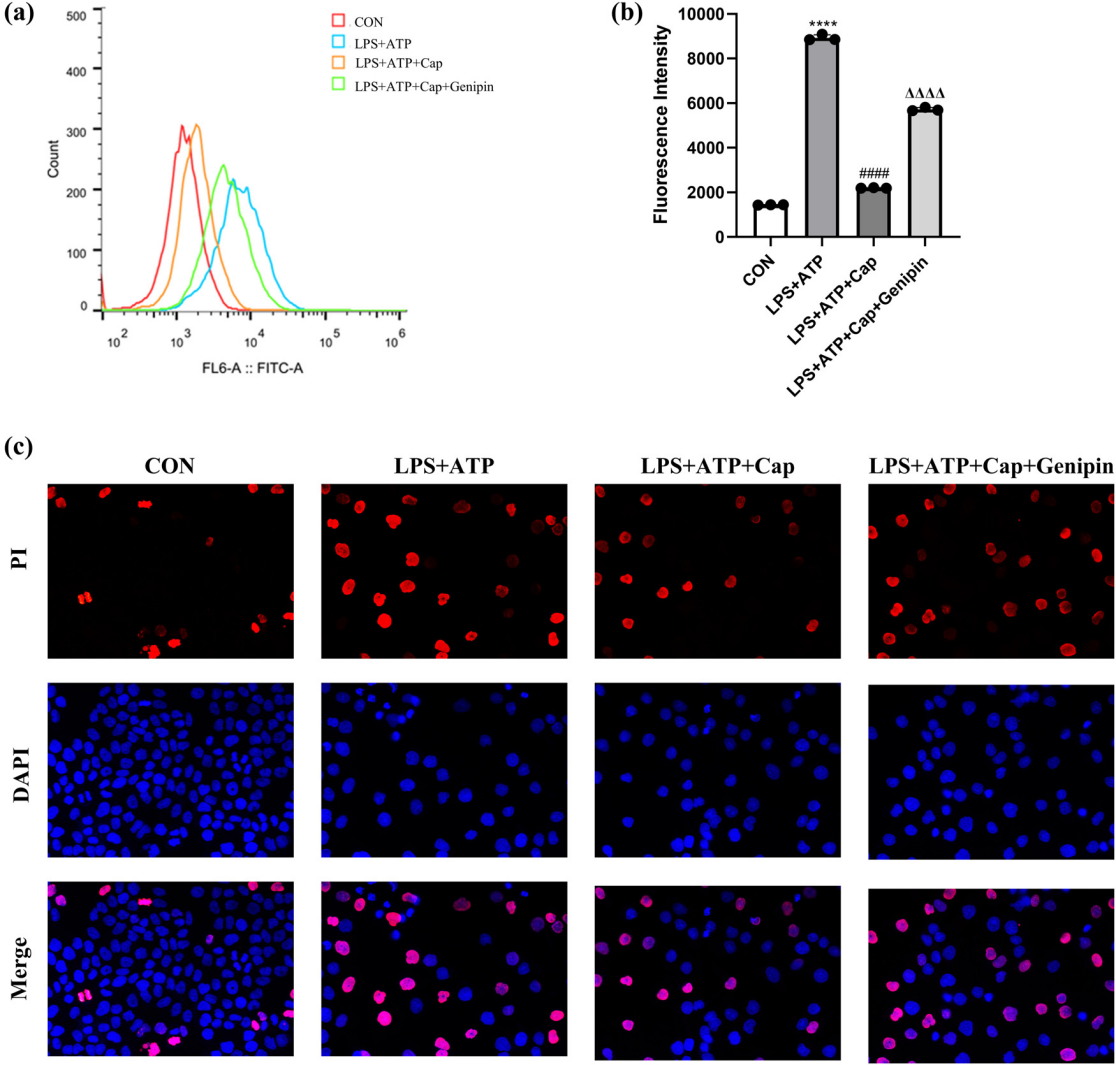
Capsaicin attenuates LPS-induced ROS generation and apoptosis in HK-2 cells. (a) ROS level was detected by flow cytometry in control, LPS + ATP, LPS + ATP + capsaicin, and LPS + ATP + capsaicin + genipin groups. (b) Quantification of ROS level in each group. Data were presented as means ± SD, ****p < 0.0001 vs CON, ####*p* < 0.0001 vs LPS + ATP, ΔΔΔΔ*p* < 0.0001 vs LPS + ATP + capsaicin. (c) Cell apoptosis was detected by PI/Hoechst staining in each group.

Increased oxidative stress and apoptosis are also prominent features of acute kidney injury [33]. Here, intracellular ROS generation was detected using dichlorodihydrofluorescein diacetate staining and flow cytometry analysis. The results showed that exposure to LPS led to a significant increase in ROS generation in HK-2 cells. However, capsaicin inhibited LPS-induced ROS production, and this effect was reversed by genipin (Figure 4a and b). Then, cell apoptosis was detected by PI staining, and red fluorescence indicates apoptotic cells. As shown in Figure 4c, the ratio of apoptotic cells increased following LPS treatment. Capsaicin treatment attenuated cell apoptosis, and this protective effect was prevented by genipin. These results indicate that capsaicin exerts a cytoprotective effect in HK-2 cells by attenuating LPS-induced ROS generation and apoptosis.

### 3.4 Capsaicin alleviates LPS-induced mitochondrial membrane potential disruption in HK-2 cells

**Figure 5:**
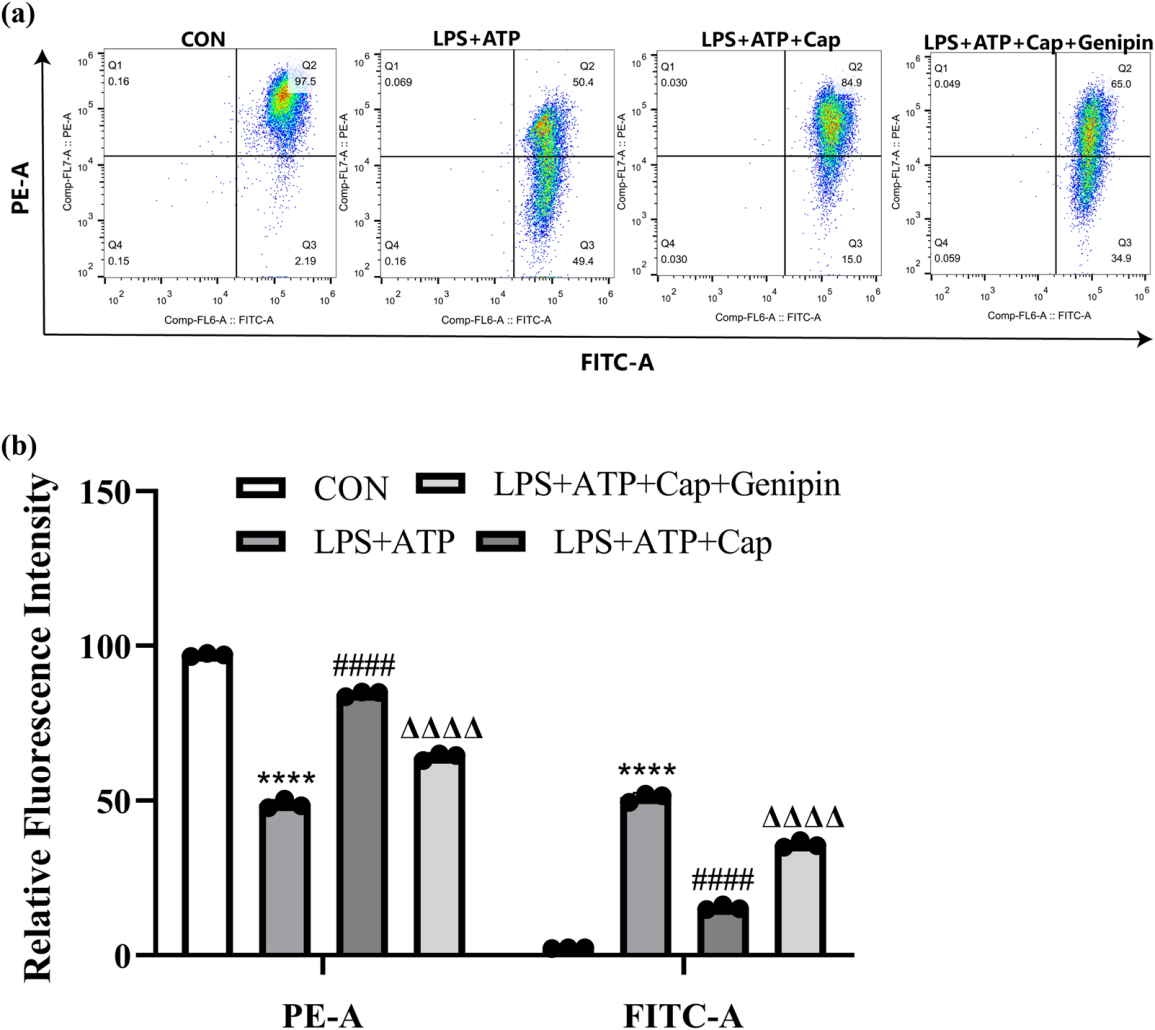
Capsaicin alleviates LPS-induced mitochondrial membrane potential disruption in HK-2 cells. (a) Mitochondrial membrane potential was detected by JC-1 staining and flow cytometry in control, LPS + ATP, LPS + ATP + capsaicin, and LPS + ATP + capsaicin + genipin groups. FITC-A (green fluorescence) indicates JC-1 monomers, and PE-A (red fluorescence) indicates JC-1 aggregates. (b) Quantification of green and red fluorescence density in each group. Data were presented as means ± SD, ****p < 0.0001 vs CON, ####*p* < 0.0001 vs LPS + ATP, ΔΔΔΔ*p* < 0.01 vs LPS + ATP + capsaicin.

As TPRV1 localizes on mitochondria and was reported to regulate mitochondrial membrane potential [34], we investigated the interplay between capsaicin and mitochondrial function. Mitochondrial membrane potential was detected by JC-1 staining and flow cytometry analysis. JC-1 is a green dye that accumulates in intact mitochondria as aggregates; JC-1 monomers emit a green fluorescence; meanwhile, JC-1 aggregates emit a red fluorescence. Results showed that LPS treatment inhibited the aggregation of JC-1, indicating that mitochondrial membrane potential was disrupted. Capsaicin treatment increased JC-1 aggregates, indicating its protective effect against LPS-induced mitochondrial membrane potential disruption. However, this effect was inhibited by genipin (Figure 5a and b), indicating that capsaicin exerts its protective effect through TRPV1/UCP2 axis.

## 4 Discussion

Acute kidney injury is characterized by death of renal tubular cells, with PCD contributing to its pathogenesis [35]. In the past decades, natural product has shown a promising effect in the prevention of acute kidney injury [36]. Here, we aim to explore the protective effect of capsaicin against acute kidney injury as well as its underlying mechanism regarding PCD, inflammation response, and mitochondrial dysfunction.

*In vitro* acute kidney injury model was established by exposure of human proximal tubular HK-2 cells to LPS as previously reported [37]. The concentrations of capsaicin used in cells can vary depending on the cell type, experimental conditions, and the specific research question being addressed. Some studies have used capsaicin at concentrations as low as 10 μM, while others have used concentrations as high as 1 mM [38,39]. Notably, it has been reported that low concentration of capsaicin (100 µM) enhances the migratory and invasive capability of SW480 and CT-26 cells, while high concentration of capsaicin (≥200 µM) inhibits cell proliferation in a dose-dependent manner [13]. In this study, the dose-dependent effect of capsaicin in HK-2 cells has been investigated in the preliminary experiment. Low concentration of capsaicin had no detectable effect on the viability of HK2 cells, while capsaicin higher than 100 µM slightly inhibited cell viability. Therefore, 100 µM of capsaicin was used in this study. It was found that capsaicin alleviated LPS cytotoxicity in HK-2 cells, which could be abolished by genipin. Capsaicin is an activator of TRPV1, and genipin is reported to inhibit UCP2 [40]. This result indicates that TRPV1/UCP2 axis might be involved in the protectiveness of capsaicin. Indeed, the results of western blot showed that capsaicin treatment up-regulated the protein levels of TRPV1 and UCP2. As a selective inhibitor of UCP2, genipin was reported to inhibit UCP2 activity [41]. However, treatment with genipin prevented capsaicin-induced up-regulation of UCP2, which is consistent with previous studies carried out in HK-2 cells [42]. Moreover, it was reported that genipin treatment decreased the mRNA level of UCP2 in NRK-52E cell, suggesting that genipin could reduce UCP2 level transcriptionally [43]. These data suggest that capsaicin protects against acute kidney injury through TRPV1/UCP2 axis.

LPS was reported to induce inflammatory response in several disease models [44,45]. In addition, inflammatory response is also a key feature of acute kidney injury [46]. Consistent with previous findings, an increased release of IL-1β and IL-18 was detected following LPS incubation. Treatment with capsaicin inhibited the release of IL-1β and IL-18, indicating that LPS-induced inflammatory response was alleviated. Previous studies showed that activated inflammation resulted in pyroptosis and apoptosis in response to various stimuli [47]. Upon activation, NLRP3 recruits ASC and caspase-1 to form the inflammasome complex, which leads to the activation of caspase-1 and the induction of pyroptosis [48]. Furthermore, activated caspase-1 cleaves pro-inflammatory cytokines, such as IL-1β and IL-18, which are released from the dying cell and contribute to the inflammatory response [49]. Not surprisingly, LPS treatment up-regulated the protein levels of caspase-1, ASC and NLRP3, and the number of PI-positive cells increased, suggesting that pyroptosis and apoptosis were activated. Treatment with capsaicin inhibited LPS-induced pyroptosis and apoptosis. Moreover, this effect was abolished by genipin, indicating that capsaicin attenuated pyroptosis and apoptosis by activating TRPV1 and UCP2.

Capsaicin has shown an anti-oxidant effect by increasing the activities of dismutase, catalase, and glutathione-S-transferase in carbon tetrachloride-induced rat liver injury [25]. Since UCP2 also plays a vital role in regulating mitochondrial function and relieving oxidative stress [50], intracellular ROS level and mitochondrial membrane potential were detected. It was found that LPS increased ROS generation and disrupted mitochondrial membrane potential in HK-2 cells, which is consistent with previous findings [33]. Treatment with capsaicin ameliorated ROS production and mitochondrial membrane potential disruption, and this effect could be prevented by genipin. Oxidative stress is an inducing factor of apoptosis, and the release of cytochrome c from the mitochondria is a key step in the intrinsic apoptotic pathway [51]. Our data confer that capsaicin protects against LPS-induced mitochondrial dysfunction through TRPV1/UCP2 axis, thereby attenuating cell apoptosis.

Notably, the application of genipin only partially reversed the protective effect of capsaicin against LPS-induced injury in HK2 cells, as indicated by results of cell viability assay, measurement of inflammatory cytokines and mitochondrial function, suggesting that other pathways might be involved in the protective effect of capsaicin. Indeed, TRPV6 was also implicated in the apoptotic action of capsaicin, which might be associated with JNK-mediated activation of Bax and p53 [52,53]. However, the role of TRPV6 in LPS-induced kidney injury has not been explored, which needs further studies.

## 5 Conclusion

Our study demonstrate that capsaicin is an effective natural product that inhibits pyroptosis and apoptosis through TRPV1/UCP2 axis. We propose the potential use of capsaicin as a novel treatment strategy for acute kidney injury.

## Supplementary Material

Supplementary Figure

## References

[1] Lameire N, Biesen WV, Vanholder R. Acute kidney injury. Lancet. 2008;372(9653):1863–5. 10.1016/S0140-6736(08)61794-8 19041789

[2] Shi L, Song Z, Li C, Deng F, Xia Y, Huang J, et al. HDAC6 inhibition alleviates ischemia- and cisplatin-induced acute kidney injury by promoting autophagy. Cells. 2022;11(24):3951. 10.3390/cells11243951 PMC9776591 36552715

[3] Brown DD, Solomon S, Lerner D, Del Rio M. Malaria and acute kidney injury. Pediatr Nephrol. 2020;35(4):603–8. 10.1007/s00467-018-4191-0 30706124

[4] Bagshaw SM, Lapinsky S, Dial S, Arabi Y, Dodek P, Wood G, et al. Acute kidney injury in septic shock: clinical outcomes and impact of duration of hypotension prior to initiation of antimicrobial therapy. Intensive Care Med. 2009;35(5):871–81. 10.1007/s00134-008-1367-2 19066848

[5] Wang Z, Wu J, Hu Z, Luo C, Wang P, Zhang Y, et al. Dexmedetomidine alleviates lipopolysaccharide-induced acute kidney injury by inhibiting p75NTR-mediated oxidative stress and apoptosis. Oxid Med Cell Longev. 2020;2020:5454210. 10.1155/2020/5454210 PMC7648709 33194004

[6] Bajwa A, Kinsey GR, Okusa MD. Immune mechanisms and novel pharmacological therapies of acute kidney injury. Curr Drug Targets. 2009;10(12):1196–204. 10.2174/138945009789753174 PMC2941672 19715538

[7] Rietschel ET, Kirikae T, Schade FU, Mamat U, Schmidt G, Loppnow H, et al. Bacterial endotoxin: molecular relationships of structure to activity and function. FASEB J. 1994;8(2):217–25. 10.1096/fasebj.8.2.8119492 8119492

[8] Mehta RL, Kellum JA, Shah SV, Molitoris BA, Ronco C, Warnock DG, et al. Acute kidney injury network: Report of an initiative to improve outcomes in acute kidney injury. Critical Care. 2007;11(2):R31. 10.1186/cc5713 PMC2206446 17331245

[9] Tewksbury JJ, Nabhan GP. Seed dispersal. Directed deterrence by capsaicin in chilies. Nature. 2001;412(6845):403–4. 10.1038/35086653 11473305

[10] Sharma SK, Vij AS, Sharma M. Mechanisms and clinical uses of capsaicin. Eur J Pharmacol. 2013;720(1-3):55–62. 10.1016/j.ejphar.2013.10.053 24211679

[11] Thongin S, Den-Udom T, Uppakara K, Sriwantana T, Sibmooh N, Laolob T, et al. Beneficial effects of capsaicin and dihydrocapsaicin on endothelial inflammation, nitric oxide production and antioxidant activity. Biomed Pharmacother. 2022;154:113521. 10.1016/j.biopha.2022.113521 36007275

[12] Clark R, Lee SH. Anticancer properties of capsaicin against human cancer. Anticancer Res. 2016;36(3):837–43. 26976969

[13] Yang J, Li TZ, Xu GH, Luo BB, Chen YX, Zhang T. Low-concentration capsaicin promotes colorectal cancer metastasis by triggering ROS production and modulating Akt/mTOR and STAT-3 pathways. Neoplasma. 2013;60(4):364–72. 10.4149/neo_2013_048 23581408

[14] Wang T, Chen Y, Li Y, Wang Z, Qiu C, Yang D, et al. TRPV1 protect against hyperglycemia and hyperlipidemia induced liver injury via OPA1 in diabetes. Tohoku J Exp Med. 2022;256(2):131–9. 10.1620/tjem.256.131 35197406

[15] Suri A, Szallasi A. The emerging role of TRPV1 in diabetes and obesity. Trends Pharmacol Sci. 2008;29(1):29–36. 10.1016/j.tips.2007.10.016 18055025

[16] Jiang XX, Liu GY, Lei H, Li ZL, Feng QP, Huang W. Activation of transient receptor potential vanilloid 1 protects the heart against apoptosis in ischemia/reperfusion injury through upregulating the PI3K/Akt signaling pathway. Int J Mol Med. 2018;41(3):1724–30. 10.3892/ijmm.2017.3338 29286076

[17] D’Arcy MS. Cell death: a review of the major forms of apoptosis, necrosis and autophagy. Cell Biol Int. 2019;43(6):582–92. 10.1002/cbin.11137 30958602

[18] Taylor RC, Cullen SP, Martin SJ. Apoptosis: controlled demolition at the cellular level. Nat Rev MolCell Biol. 2008;9(3):231–41. 10.1038/nrm2312 18073771

[19] Xu X, Lai Y, Hua ZC. Apoptosis and apoptotic body: disease message and therapeutic target potentials. Biosci Rep. 2019;39:1. 10.1042/BSR20180992 PMC6340950 30530866

[20] McKenzie BA, Mamik MK, Saito LB, Boghozian R, Monaco MC, Major EO, et al. Caspase-1 inhibition prevents glial inflammasome activation and pyroptosis in models of multiple sclerosis. Proc Natl Acad Sci U S A. 2018;115(26):E6065–E74. 10.1073/pnas.1722041115 PMC6042136 29895691

[21] Fernandes-Alnemri T, Wu J, Yu JW, Datta P, Miller B, Jankowski W, et al. The pyroptosome: a supramolecular assembly of ASC dimers mediating inflammatory cell death via caspase-1 activation. Cell Death Differ. 2007;14(9):1590–604. 10.1038/sj.cdd.4402194 PMC3345951 17599095

[22] Wu Y, Zhang J, Yu S, Li Y, Zhu J, Zhang K, et al. Cell pyroptosis in health and inflammatory diseases. Cell Death Discov. 2022;8(1):191. 10.1038/s41420-022-00998-3 PMC8995683 35411030

[23] Xia W, Li Y, Wu M, Jin Q, Wang Q, Li S, et al. Gasdermin E deficiency attenuates acute kidney injury by inhibiting pyroptosis and inflammation. Cell Death Dis. 2021;12(2):139. 10.1038/s41419-021-03431-2 PMC7862699 33542198

[24] Park SG, Yon JM, Lin C, Gwon LW, Lee JG, Baek IJ, et al. Capsaicin attenuates spermatogenic cell death induced by scrotal hyperthermia through its antioxidative and anti-apoptotic activities. Andrologia. 2017;49(5). 10.1111/and.12656. 27401946

[25] Hassan MH, Edfawy M, Mansour A, Hamed AA. Antioxidant and antiapoptotic effects of capsaicin against carbon tetrachloride-induced hepatotoxicity in rats. Toxicol Ind Health. 2012;28(5):428–38. 10.1177/0748233711413801 21859771

[26] Liu J, Li G, Xie WJ, Wang L, Zhang R, Huang KS, et al. Lipopolysaccharide stimulates surfactant protein-A in human renal epithelial HK-2 cells through upregulating toll-like receptor 4 dependent MEK1/2-ERK1/2-NF-kappaB pathway. Chin Med J (Engl). 2017;130(10):1236–43. 10.4103/0366-6999.205853 PMC5443031 28485325

[27] Tan C, Gu J, Li T, Chen H, Liu K, Liu M, et al. Inhibition of aerobic glycolysis alleviates sepsis‑induced acute kidney injury by promoting lactate/Sirtuin 3/AMPK‑regulated autophagy. Int J Mol Med. 2021;47(3):19. 10.3892/ijmm.2021.4852 PMC7849980 33448325

[28] Qiongyue Z, Xin Y, Meng P, Sulin M, Yanlin W, Xinyi L, et al. Post-treatment with irisin attenuates acute kidney injury in sepsis mice through anti-ferroptosis via the SIRT1/Nrf2 pathway. Front Pharmacol. 2022;13:857067. 10.3389/fphar.2022.857067 PMC8970707 35370723

[29] Li L, Chen J, Ni Y, Feng X, Zhao Z, Wang P, et al. TRPV1 activation prevents nonalcoholic fatty liver through UCP2 upregulation in mice. Pflugers Arch. 2012;463(5):727–32. 10.1007/s00424-012-1078-y 22395410

[30] Sun J, Pu Y, Wang P, Chen S, Zhao Y, Liu C, et al. TRPV1-mediated UCP2 upregulation ameliorates hyperglycemia-induced endothelial dysfunction. Cardiovasc Diabetol. 2013;12:69. 10.1186/1475-2840-12-69 PMC3644255 23607427

[31] Wang Y, Zhang H, Chen Q, Jiao F, Shi C, Pei M, et al. TNF-alpha/HMGB1 inflammation signalling pathway regulates pyroptosis during liver failure and acute kidney injury. Cell Prolif. 2020;53(6):e12829. 10.1111/cpr.12829 PMC7309595 32419317

[32] Jayakumar S, Jennings S, Halvorsrud K, Clesse C, Yaqoob MM, Carvalho LA, et al. A systematic review and meta-analysis of the evidence on inflammation in depressive illness and symptoms in chronic and end-stage kidney disease. Psychol Med. 2022;1–13. 10.1017/S0033291722003099. 36254747

[33] Jha JC, Banal C, Chow BS, Cooper ME, Jandeleit-Dahm K. Diabetes and kidney disease: Role of oxidative stress. Antioxid Redox Signal. 2016;25(12):657–84. 10.1089/ars.2016.6664 PMC5069735 26906673

[34] Hurt CM, Lu Y, Stary CM, Piplani H, Small BA, Urban TJ, et al. Transient Receptor Potential Vanilloid 1 Regulates Mitochondrial Membrane Potential and Myocardial Reperfusion Injury. J Am Heart Assoc. 2016;5(9):e003774. 10.1161/JAHA.116.003774 PMC5079036 27671317

[35] Lin Q, Li S, Jiang N, Jin H, Shao X, Zhu X, et al. nhibiting NLRP3 inflammasome attenuates apoptosis in contrast-induced acute kidney injury through the upregulation of HIF1A and BNIP3-mediated mitophagy. Autophagy. 2021;17(10):2975–90. 10.1080/15548627.2020.1848971 PMC8525960 33345685

[36] Kang HG, Lee HK, Cho KB, Park SI. A review of natural products for prevention of acute kidney injury. Medicina (Kaunas). 2021;57(11):1266. 10.3390/medicina57111266 PMC8623373 34833485

[37] Hu M, Wei J, Yang L, Xu J, He Z, Li H, et al. Linc-KIAA1737-2 promoted LPS-induced HK-2 cell apoptosis by regulating miR-27a-3p/TLR4/NF-kappaB axis. J Bioenerg Biomembr. 2021;53(4):393–403. 10.1007/s10863-021-09897-1 PMC8360891 34076840

[38] Hwang MK, Bode AM, Byun S, Song NR, Lee HJ, Lee KW, et al. Cocarcinogenic effect of capsaicin involves activation of EGFR signaling but not TRPV1. Cancer Res. 2010;70(17):6859–69. 10.1158/0008-5472.CAN-09-4393 20660715

[39] Mori A, Lehmann S, O’Kelly J, Kumagai T, Desmond JC, Pervan M, et al. Capsaicin, a component of red peppers, inhibits the growth of androgen-independent, p53 mutant prostate cancer cells. Cancer Res. 2006;66(6):3222–9. 10.1158/0008-5472.CAN-05-0087 16540674

[40] Cho YS. Genipin, an inhibitor of UCP2 as a promising new anticancer agent: a review of the literature. Int J Mol Sci. 2022;23(10):5637. 10.3390/ijms23105637 PMC9147402 35628447

[41] Zhang CY, Parton LE, Ye CP, Krauss S, Shen R, Lin CT, et al. Genipin inhibits UCP2-mediated proton leak and acutely reverses obesity- and high glucose-induced beta cell dysfunction in isolated pancreatic islets. Cell Metab. 2006;3(6):417–27. 10.1016/j.cmet.2006.04.010 16753577

[42] Feng C, Anger EE, Zhang X, Su S, Su C, Zhao S, et al. Protective effects of mitochondrial uncoupling protein 2 against aristolochic acid i-induced toxicity in HK-2 cells. Int J Mol Sci. 2022;23(7):3674. 10.3390/ijms23073674 PMC8998172 35409033

[43] Chen XL, Tang WX, Tang XH, Qin W, Gong M. Downregulation of uncoupling protein-2 by genipin exacerbates diabetes-induced kidney proximal tubular cells apoptosis. Ren Fail. 2014;36(8):1298–303. 10.3109/0886022X.2014.930650 24964191

[44] You Z, Yang Z, Cao S, Deng S, Chen Y. The Novel KLF4/BIG1 Regulates LPS-mediated Neuro-inflammation and Migration in BV2 Cells via PI3K/Akt/NF-kB Signaling Pathway. Neuroscience. 2022;488:102–11. 10.1016/j.neuroscience.2022.01.014 35090882

[45] Shen P, Ji S, Li X, Yang Q, Xu B, Wong CKC, et al. LPS-Induced Systemic Inflammation Caused mPOA-FSH/LH Disturbance and Impaired Testicular Function. Front Endocrinol (Lausanne). 2022;13:886085. 10.3389/fendo.2022.886085 PMC9259990 35813649

[46] Ren Q, Guo F, Tao S, Huang R, Ma L, Fu P. Flavonoid fisetin alleviates kidney inflammation and apoptosis via inhibiting Src-mediated NF-kappaB p65 and MAPK signaling pathways in septic AKI mice. Biomed Pharmacother. 2020;122:109772. 10.1016/j.biopha.2019.109772 31918290

[47] Kadono K, Kageyama S, Nakamura K, Hirao H, Ito T, Kojima H, et al. Myeloid Ikaros-SIRT1 signaling axis regulates hepatic inflammation and pyroptosis in ischemia-stressed mouse and human liver. J Hepatol. 2022;76(4):896–909. 10.1016/j.jhep.2021.11.026 PMC9704689 34871625

[48] Zhou Z, Li C, Bao T, Zhao X, Xiong W, Luo C, et al. Exosome-shuttled miR-672-5p from anti-inflammatory microglia repair traumatic spinal cord injury by inhibiting AIM2/ASC/Caspase-1 signaling pathway mediated neuronal pyroptosis. J Neurotrauma. 2022;39(15–16):1057–74. 10.1089/neu.2021.0464 35243913

[49] Guo Z, Tan B, Wang J, Tang W, Pei L, Chen Y, et al. Activation of alpha-7 nicotinic acetylcholine receptor attenuates cardiac inflammation through NLRP3/caspase-1/IL-18 pathway. Biochem Genet. 2022;60(4):1333–45. 10.1007/s10528-021-10162-8 34988776

[50] Pierelli G, Stanzione R, Forte M, Migliarino S, Perelli M, Volpe M, et al. Uncoupling protein 2: A key player and a potential therapeutic target in vascular diseases. Oxid Med Cell Longev. 2017;2017:7348372. 10.1155/2017/7348372 PMC5661070 29163755

[51] Xie H, Song L, Katz S, Zhu J, Liu Y, Tang J, et al. Electron transfer between cytochrome c and microsomal monooxygenase generates reactive oxygen species that accelerates apoptosis. Redox Biol. 2022;53:102340. 10.1016/j.redox.2022.102340 PMC9130584 35609401

[52] Chow J, Norng M, Zhang J, Chai J. TRPV6 mediates capsaicin-induced apoptosis in gastric cancer cells--Mechanisms behind a possible new “hot” cancer treatment. Biochim Biophys Acta. 2007;1773(4):565–76. 10.1016/j.bbamcr.2007.01.001 17292493

[53] Lau JK, Brown KC, Dom AM, Witte TR, Thornhill BA, Crabtree CM, et al. Capsaicin induces apoptosis in human small cell lung cancer via the TRPV6 receptor and the calpain pathway. Apoptosis. 2014;19(8):1190–201. 10.1007/s10495-014-1007-y PMC4072851 24878626

