# Supplementary material for: Inhibition of pyroptosis and apoptosis by capsaicin protects against LPS-induced acute kidney injury through TRPV1/UCP2 axis in vitro
Source: Open Life Sci. 2023 Jul 29;18(1):20220647. doi: 10.1515/biol-2022-0647 (PMC10389676; doi:10.1515/biol-2022-0647)
Supplement: Supplementary Figure [file biol-2022-0647-sm.pdf]

Supplementary material

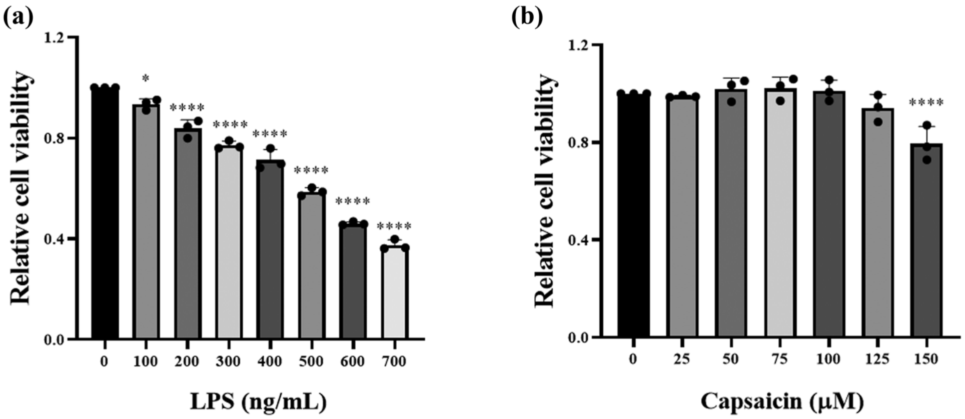

Figure S1: Cytotoxicity of LPS and Capsaicin in HK-2 cells. HK-2 cells were treated with different concentrations of LPS (a) or Capsaicin (b). Cell viability was detected by CCK8 assay. Data were presented as the means ± SD, *$p < 0.05$, ****$p < 0.0001$.
